# Utility of Orthosis in the Conservative Treatment of Proximal Phalanx Fractures: A Preliminary Study in Healthy Participants

**DOI:** 10.7759/cureus.73675

**Published:** 2024-11-14

**Authors:** Risako Nakanishi, Shuichi Sasaki, Naoto Kamide, Sohei Takamori, Yoshito Nakajima, Satomi Matsumoto, Kenji Onuma, Koji Sukegawa, Yuya Otake, Naonobu Takahira

**Affiliations:** 1 Department of Rehabilitation, Yokohama Minami Kyosai Hospital, Yokohama, JPN; 2 Graduate School of Medical Sciences, Kitasato University, Sagamihara, JPN; 3 Division of Rehabilitation, Kitasato University Hospital, Sagamihara, JPN; 4 School of Allied Health Sciences, Kitasato University, Sagamihara, JPN; 5 Sports Medicine Center, Shonan Kamakura General Hospital, Kamakura, JPN; 6 Department of Radiology, Yokohama Minami Kyosai Hospital, Yokohama, JPN; 7 Clinical Research Center, Yokohama Minami Kyosai Hospital, Yokohama, JPN; 8 Department of Orthopedic Surgery, Kitasato University School of Medicine, Sagamihara, JPN; 9 Department of Clinical Anatomy, Research and Development Center for Medical Education, Kitasato University School of Medicine, Sagamihara, JPN; 10 Graduate School of Medical, Kitasato University, Sagamihara, JPN

**Keywords:** cast immobilization, conservative treatment, fine motor movements, orthosis, proximal phalanx fractures

## Abstract

Background: The standard treatment for the conservative management of a proximal phalanx fracture of the little finger involves immobilizing the fracture site with a cast. However, cast immobilization presents challenges in maintaining hygiene during treatment and restricts the fine motor movements of the fingers. In this study, we developed a removable orthosis that immobilizes only the ring and little fingers. We examined the splint's effectiveness in joint immobilization and its impact on fine motor movements of the fingers.

Method: Twenty healthy adults were included in this study conducted at the Yokohama Minami Kyosai Hospital, Yokohama, Japan. While wearing a cast and an orthosis, the range of motion of the metacarpophalangeal (MP) joints of the index and little fingers was measured using X-ray fluoroscopy. The Purdue Pegboard Test evaluated fine motor skills, handwriting tasks, and chopstick use tests. We statistically compared the range of motion and fine motor skills of the MP joints in the cast and orthosis.

Results: The range of motion of the MP joint in the index finger was significantly greater with the orthosis than with the cast (p < 0.001). In contrast, in the little finger, the cast allowed a greater range of motion than the orthosis (p < 0.077). Additionally, regarding fine motor skills, in the chopstick task, the orthosis showed significantly better performance than the cast.

Conclusion: The developed orthosis, which immobilized only the ring and little fingers, provided sufficient stability to the little finger while maintaining the index finger’s mobility. Additionally, it imposed minimal restrictions on fine motor movements and the use of chopsticks.

## Introduction

In a survey conducted in the United States, finger injuries accounted for 38.4% of upper limb injuries and, therefore, the most common [1]. Among the fractures caused by finger injuries, fractures of the little finger have the highest incidence. Furthermore, fractures of the distal and proximal phalanges in the dominant hand have high occurrence rates [2]. Conservative therapy is typically selected for the management of relatively stable fractures that do not require open surgery [3].

Burkhalter et al. reported in 1984 on the conservative treatment of proximal phalanx fractures involving the immobilization of the metacarpophalangeal (MP) joint in a flexed position, extending from the proximal interphalangeal (PIP) joint to the forearm [4]. In Japan in 1991, Ishiguro introduced the ‘knuckle cast’ method, which involves immobilizing only the MP joint in a flexed position, with active movement of the PIP and distal interphalangeal (DIP) joints, without immobilizing the wrist [5]. In 2011, Figl et al. reported that by immobilizing the MP joint in a flexed position while allowing finger movement, fractures could be treated without residual restrictions in the range of motion (ROM) [6].

These conservative treatments yield good outcomes with a low risk of MP joint flexion restrictions [4-6]. However, these treatments also involve immobilizing the finger joints, and the cast must be worn until the fracture heals. Consequently, finger movements are restricted during treatment, which can interfere with activities of daily living (ADL). Additionally, if there is dirtiness due to sweating, hygiene maintenance is difficult in the immobilized area because of lack of washing.

In recent years, treatments using orthosis that are similar to casts have been reported [7]. Successful outcomes have been reported for the conservative treatment of proximal phalanx fractures of the little finger using buddy taping, which immobilizes only the ring and little fingers together [8]. Therefore, an orthosis that immobilizes only the ring and little fingers could also be effective for the conservative management of proximal phalanx fractures of the little finger. However, the effectiveness of such an orthosis on joint immobilization and its impact on fine motor skills have not been sufficiently investigated.

In this study, we developed a removable orthosis that immobilizes only the ring and little fingers for the conservative treatment of common proximal phalanx fractures of the little finger. The purpose of this study was to compare and evaluate joint immobilization and fine motor skills between conventional and newly developed orthosis and to assess the utility of the developed orthosis.

## Materials and methods

This was a study conducted at theDepartment of Rehabilitation, Yokohama Minami Kyosai Hospital, Yokohama, Japan, to evaluate the effectiveness of a removable orthosis in a cohort of healthy subjects. The participants were provided with both written and verbal explanations of the purpose and methods, and written informed consent was obtained from all. The study was conducted in compliance with the Declaration of Helsinki of the World Medical Association and the Ethical Guidelines for Medical Research Involving Human Subjects of the Ministry of Health, Labour and Welfare, and the Ministry of Education, Culture, Sports, Science, and Technology, Japan. The study was approved by the Ethics Committee of Yokohama Minami Kyosai Hospital (approval number: 1-20-10-7). It was registered with the University hospital Medical Information Network (UMIN) Clinical Trials Registry (UMIN: 000051579).

Participants

Twenty healthy adults were recruited as volunteers. The volunteers comprised hospital staff including nurses, laboratory technicians, and therapists, who consented after being informed of the study's purpose and potential adverse effects. The inclusion criteria were as follows: right-handedness, no history of finger injuries and congenital finger deformities or deficiencies, no restrictions in the ROM of the finger or muscle weakness, and age 20 or older. The exclusion criteria were pregnancy, left-handedness, restricted ROM of the fingers, or muscle weakness. The researchers confirmed the criteria through in-person assessments.

Method of preparation of the orthosis

**Figure 1:**
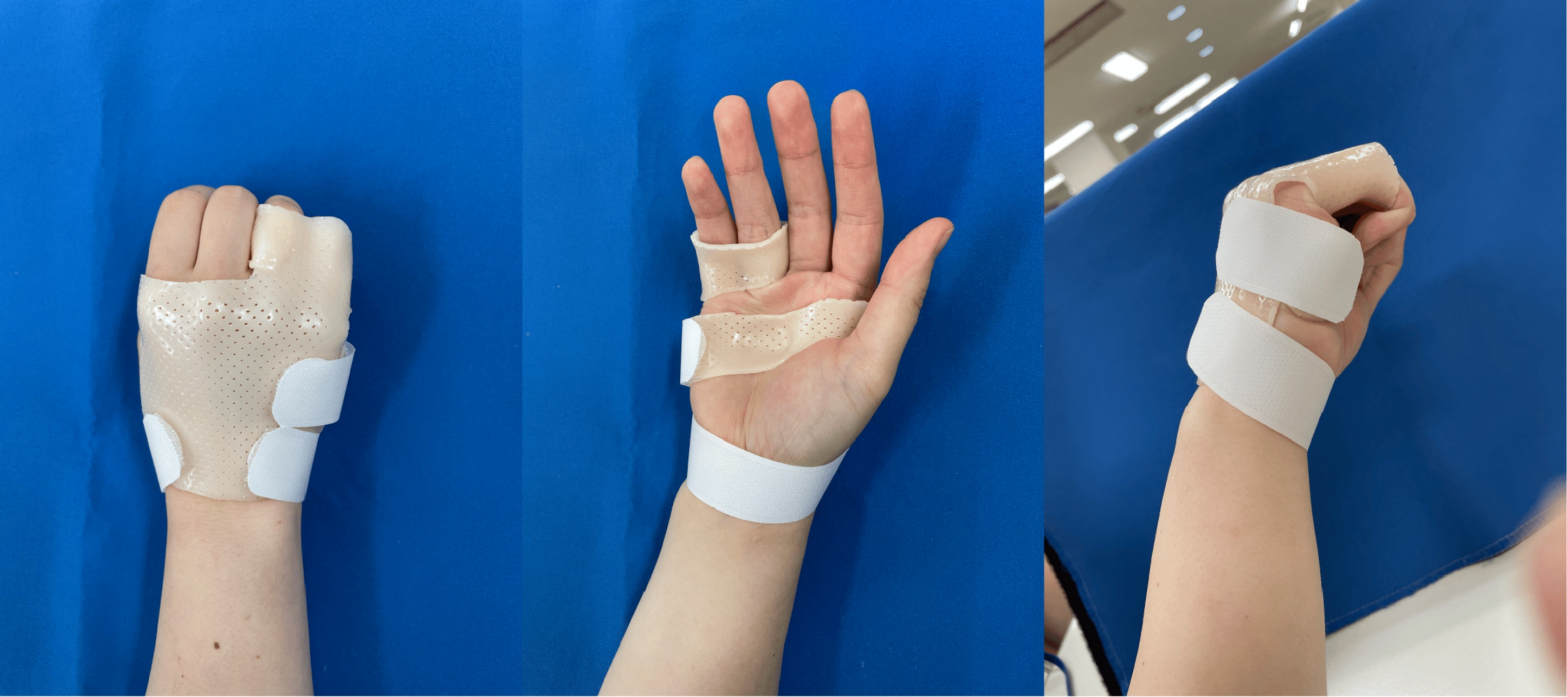
Orthosis developed in this study that fixes the ring and little fingers The MP joint was fixed in flexion in the ring and little fingers. In contrast, the thumb, index, and middle fingers were not fixed and were thereby free to move.

The orthosis (Figure 1) was created by positioning the MP joints of the ring and little fingers in a flexed position and securing the proximal phalanges of these fingers together in a single loop, with care being taken not to restrict the movement of the PIP joint. In addition, the design of the orthosis allowed movements of the MP, PIP, and DIP joints of the index and middle fingers. The palmar side of the orthosis conformed to the transverse arch of the hand while the wrist was left unfixed. The orthosis was made from a thermoplastic material (Orfit NS 2.0 mm, Pacific Supply Co., Ltd, Osaka, Japan), and the same occupational therapist carried out the fabrication throughout the study.

Assessment of the finger joint immobilization

The casting was performed by an orthopedic surgeon with 20 years of experience, following the method described by Ishiguro [9]. The cast was briefly applied to immobilize the MP joints of the index and the little fingers in a flexed position of 70-90°. Care was taken to ensure that the cast did not interfere with the PIP joints or wrist. Additionally, special attention was paid to prevent the cast from affecting the thumb.

**Figure 2:**
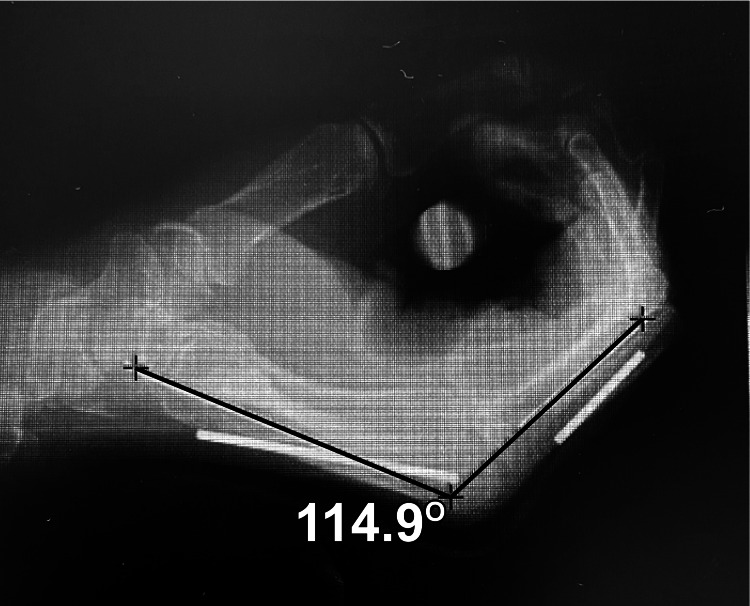
Measurement methods for the maximum range of motion at the index and little fingers Using a copper wire attached just above the basal and metacarpal bones of the index and little fingers, flexion and extension exercises were performed twice for five seconds. Using an X-ray fluoroscopy system, the maximum range of motion in flexion and extension of the index and little fingers during the exercise was measured.

The ROM of the MP joints of the index and little fingers was measured using an X-ray fluoroscopy device (VersiFlex SF-VA2000FP1; Hitachi Medical Corporation, Tokyo, Japan) to evaluate finger joint immobilization. Concurrently, the participants wore either the cast or the orthosis. During the wearing of either the cast or the orthosis, the participants performed flexion and extension movements of the fingers at a speed of two movements per five seconds, with images captured three times per second (Figure 2). To accurately identify the metacarpal and proximal phalangeal bones of the index and little fingers during the measurement of the MP joint mobility, copper wires (3 mm in diameter) were affixed to the metacarpal and proximal phalangeal bones of these fingers (Figure 2). Fifteen images were captured over five seconds, and the maximum flexion and extension angles of the MP joints of the index and little fingers were measured. The maximum flexion and extension angles determined the maximum range of motion. The Centricity Digital Imaging and Communications in Medicine (DICOM) viewer (AW Z 800 HW4.6SW) were used to measure the MP joint angles. A researcher conducted the measurements twice to ensure reproducibility, and the average of these measurements was used for the statistical analysis.

Comparison of fine motor skills

**Figure 3:**
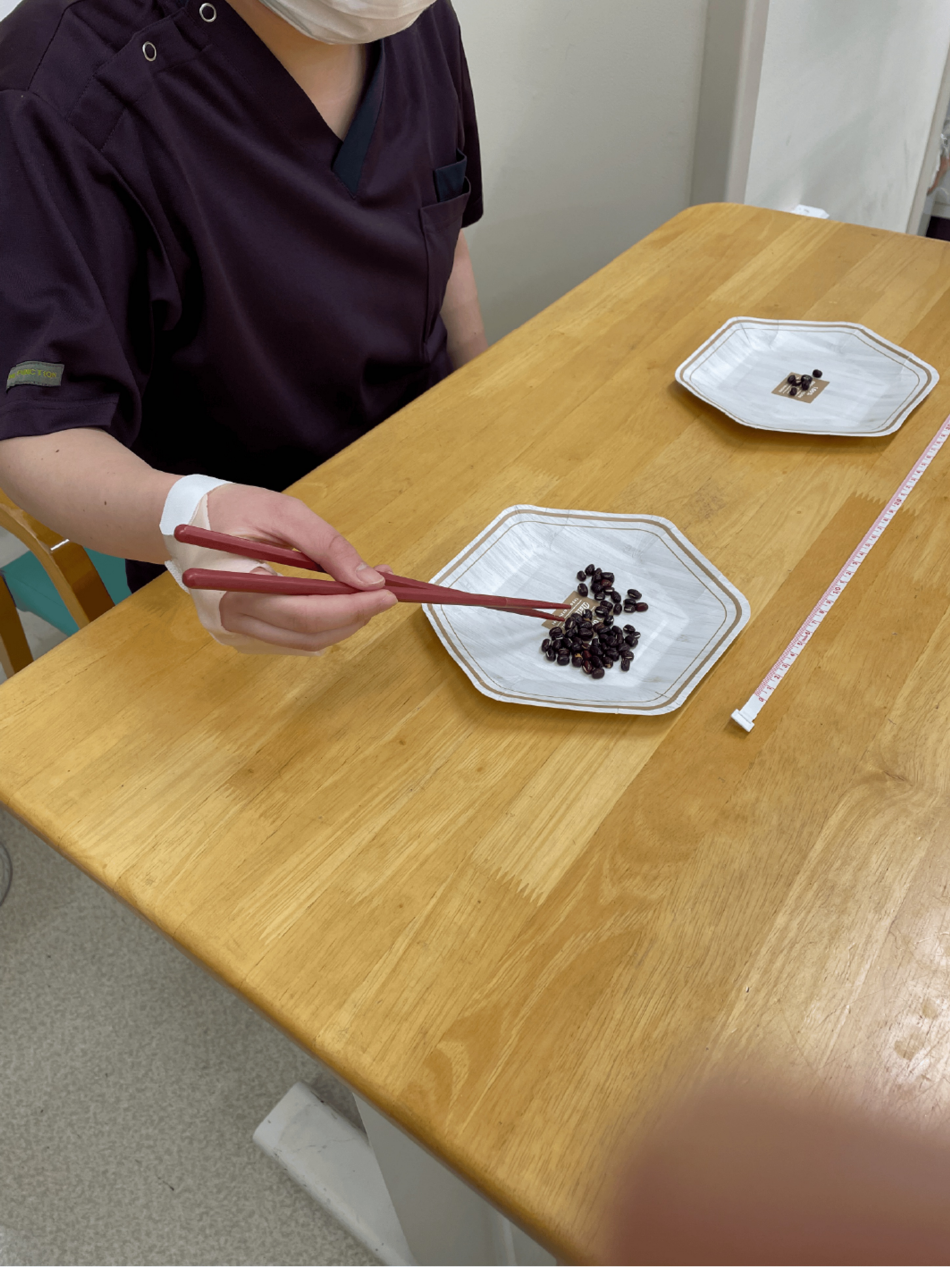
Chopstick test This test measures the number of beans carried with chopsticks from one plate to the other placed at a distance of 30 cm apart in 30 seconds.

Fine motor skills were compared between participants wearing either the cast or the orthosis using the Purdue Pegboard Test (PPT) and handwriting and chopstick use task. Participants were instructed to insert pins into holes for 30 seconds for the PPT. This task was repeated three times. The average number of pins successfully inserted was recorded. Based on a previous study [10], for the handwriting task, the participants were instructed to write a predetermined sentence as neatly as possible on a specified sheet of paper using a pencil. The time taken to complete the writing was measured. Two plates were placed 30 cm apart, with one filled with red beans for the chopstick use task. The participants used chopsticks to transfer the red beans from one plate to another within 30 seconds, and the number of beans transferred was recorded (Figure 3) [11,12]. All participants used the same set of chopsticks. For the evaluation of fine motor skills, all participants used the same chair and desk. The papers used for the PPT and handwriting tasks, as well as the plates for the chopstick use task, were placed in identical positions for each participant to ensure consistency.

Statistical analysis

A linear regression model was used to analyze the differences in the ROM of the index and little fingers between the cast and orthosis groups. The ROM was treated as the dependent variable, the condition (cast or orthosis) as the independent variable, and age, sex, and grip strength as covariates. A paired t-test was used to compare the functional performance of each finger under the cast and orthosis. Statistical significance was set at 5%, and all statistical analyses were performed using R programming language and environment (R version 4.2.2; R Foundation for Statistical Computing, Vienna, Austria).

## Results

The average age of the 20 participants (5 male and 15 female participants) was 43.5 ± 10.7 years. The average grip strength for male and female volunteers was 42.0 ± 5.2 kg and 26.4 ± 5.3 kg, respectively.

**Figure 4:**
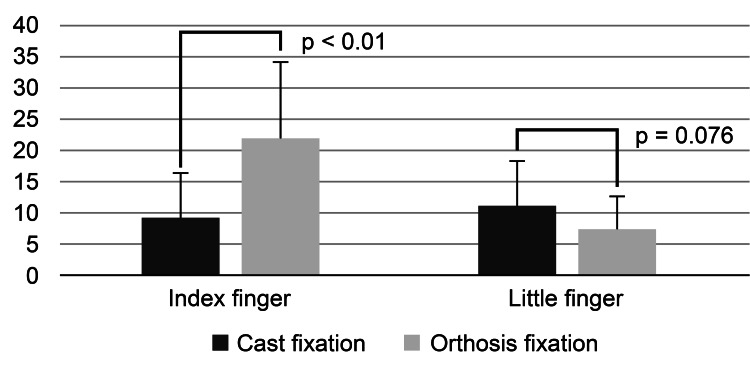
Comparison of the range of motion in index and little fingers under two conditions (cast and orthosis)

The average maximum ROM for the flexion-extension movement of the MP joint when using the cast was 9.1 ± 7.2 and 11.0 ± 7.3 degrees for the index and the little finger, respectively. Compared with the cast, the average ROM with the orthosis was 21.9 ± 12.1 and 7.3 ± 5.2 degrees for the index finger and the little finger. The maximum ROM for the flexion-extension movement of the index finger after adjusting for age, sex, and grip strength was significantly greater with the orthosis than with the cast (Figure 4, Table 1). Conversely, the maximum range of the little finger was smaller with the orthosis than with the cast; however, the difference was not statistically significant (Table 2 and Figure 4).

**Table 1:** Comparison of the range of motion in the index finger under two conditions (cast and orthosis) adjusted by potential confounders The dependent variable was the range of motion in the index finger; Coefficients were indicated in unstandardized coefficients SE: standard error

	Coefficients	SE	t-value	p-value
Age (years)	0.11	0.2	0.561	0.579
Sex (male)	6.45	6.31	1.022	0.314
Grip strength (kg)	-0.28	0.36	-0.779	0.441
Fixation condition (orthosis)	12.77	3.29	3.882	<0.001

**Table 2:** Comparison of the range of motion in little finger under two conditions (cast and orthosis) adjusted by potential confounders The dependent variable was the range of motion in the little finger; Coefficients were indicated in unstandardized coefficients SE: standard error

	Coefficients	SE	t-value	p-value
Age (years)	-0.07	0.12	-0.591	0.558
Sex (male)	-1.12	3.82	-0.294	0.771
Grip strength (kg)	0.26	0.22	1.179	0.247
Fixation condition (orthosis)	-3.64	1.99	-1.827	0.077

There was no significant difference in the performance of the cast and orthosis conditions in the PPT regarding fine motor skills. However, the participants completed the handwriting task significantly faster with a cast than with an orthosis. In the chopstick task, participants tended to perform better with the orthosis than with the cast (Table 3).

**Table 3:** Hand function performance tests under two conditions (cast and orthosis)

	Cast	Splint	t-value	p-value
	Mean (SD)			
Purdue Pegboard Test (number of pieces carried)	14.1 (1.5)	14.3 (1.8)	0.646	0.526
Chopsticks test (number of beans carried)	16.7 (6.1)	19.7 (5.1)	2.058	0.054
Writing test (number of seconds taken)	24.4 (5.8)	26.3 (7.0)	2.988	0.008

## Discussion

In this study, we evaluated the effectiveness of a newly developed removable orthosis designed to immobilize only the ring and little fingers while focusing on joint stability and fine motor skills. The results showed that when comparing the cast immobilization to that of the newly developed orthosis, the orthosis had a smaller maximum ROM in the MP joint of the little finger. This suggested that the orthosis provided a comparable stability to the cast. However, open surgery is generally preferred for challenging proximal phalangeal fractures that are difficult to treat conservatively. Conservative therapy may be appropriate for relatively stable fractures [4]. In addition, some reports have suggested that conservative treatment is feasible for fractures involving a single finger [13]. Furthermore, even for fractures that typically require surgical intervention, a prospective study showed that 90% of cases had favorable outcomes with conservative treatment [14]. Therefore, many cases of proximal phalangeal fractures of the little finger can be conservatively treated. The orthosis developed in this study may be a valuable option for the conservative treatment of proximal phalanx fractures in the little finger, thereby offering a significant alternative.

Successful healing with immobilization using an orthosis rather than a cast has been reported in the conservative management of proximal phalanx fractures [15]. However, it was unclear whether the splint adequately stabilized the joint. There was insufficient information regarding the safety of this treatment. This present study demonstrated that an orthosis immobilizes only the MP joints of the ring and little fingers and tends to provide a reduced maximum ROM in the MP joint of the little finger compared to a cast that immobilizes all four fingers except the thumb. This finding suggested that the orthosis offers a level of stability comparable to that of a cast. In contrast, for the index finger, the orthosis allowed a greater ROM than the cast. The orthosis used in this study did not hinder the movement of the index and middle fingers that were not immobilized, potentially making it effective in preventing contractures associated with immobilization during treatment.

Regarding fine motor skills, chopstick use tended to be better with the orthosis than with the cast. This improvement was probably because of the involvement of the thumb, index finger, and middle finger joint movements, which are essential for chopstick use [16]. The results of this study showed that the orthosis allowed for a significantly greater ROM in the index finger than did the cast. The maximum ROM was not evaluated for the thumb and middle fingers as the orthosis did not immobilize these digits. Therefore, it is believed that during treatment, orthosis imposes fewer restrictions on eating activities, a part of ADL movement. On the other hand, for the writing tasks, the cast performed better than the orthosis. This observation suggested that the restricted maximum ROM in the little finger with the orthosis compared to that with the cast may have impacted writing performance. There was no significant difference in PPT scores between the orthosis and cast groups. Previous studies have noted that there may be no correlation between ADL and the PPT results [17]. In other words, the pinching movements evaluated using the PPT were relatively simple, leading to a ceiling effect in healthy individuals. These findings indicated that in the context of conservative treatment of proximal phalangeal fractures, it is crucial to choose a treatment method that does not hinder the most important tasks in a patient’s life. The orthosis developed in this study may offer available treatment options tailored to the specific needs of patients. Moreover, a notable advantage of immobilization by an orthosis is its ability to facilitate hand hygiene. The inability to remove the cast after immobilization makes good hand hygiene maintenance challenging. In contrast, the orthosis developed in this study allowed easy removal, enabling patients to wash their hands. The ability to maintain hand hygiene is expected to reduce stress during treatment [18]

The mechanism by which the orthosis demonstrated effective immobilization of the little finger in this study could not be clarified based on the data. However, one possible explanation is the anatomical characteristics of fingers. In the conservative treatment of proximal phalanx fractures using a cast, the MP joint is typically immobilized in a flexed position of 70-90°. This flexed position of the MP joint causes the dorsal extensor aponeurosis to stretch and cover approximately two-thirds of the proximal phalanx, thereby functioning as a tension band [19]. The key to the conservative treatment of proximal phalanx fractures lies in immobilizing the MP joint in a flexed position, allowing for stability of the fracture site through controlled flexion and extension of the fingers [20]. The flexion of the MP joint is crucial because it maximizes tension on the collateral ligaments, which stabilizes the MP joint [21]. In this study, the developed orthosis immobilized the ring and little fingers in the flexed position of the MP joint. Therefore, it is presumed that with the immobilization of these two fingers, the same stabilizing effect was achieved on the MP joint, similar to that mentioned previously. Moreover, the stability provided by the MP joint in the flexion is enhanced further by the deep transverse metacarpal ligament and palmar plate [22]. The deep transverse metacarpal ligament runs between the heads of the metacarpal bones, creating a strong connection. This strong connection may allow for the stabilization of the little finger when the MP joints of the index and middle fingers are not fixed. Another factor to consider is the difference in fixation methods between the orthosis and cast. Generally, a padding material is used in cast applications [23]. This method was used in this study. The padding material may cause some looseness during cast immobilization. In contrast, the orthosis was molded directly onto the skin with close adherence and worn without any padding. The difference in the immobilization methods may have influenced the results.

In this study, there were some limitations. First, the developed orthosis was specifically designed for proximal phalanx fractures of the little finger. Therefore, it is uncertain whether these results can be applied to proximal phalanx fractures of other fingers. Second, this study involved healthy individuals; therefore, treatment outcomes may differ when applied to patients with actual fractures. Future research should include an evaluation of treatment outcomes with orthosis in real cases and conduct randomized controlled trials to validate its effectiveness.

## Conclusions

Considering proximal phalanx fractures of the little finger, the orthosis developed in this study for immobilization of the ring and little fingers demonstrated sufficient immobilization of the little finger while preserving the mobility of the index finger. Additionally, it was noted to have minimal impact on fine motor skills, such as that while using chopsticks. Therefore, this orthosis is a useful treatment option for conservative treatment of proximal phalanx fractures of the little finger.
